# Replication Termination: Containing Fork Fusion-Mediated Pathologies in *Escherichia coli*

**DOI:** 10.3390/genes7080040

**Published:** 2016-07-25

**Authors:** Juachi U. Dimude, Sarah L. Midgley-Smith, Monja Stein, Christian J. Rudolph

**Affiliations:** Division of Biosciences, College of Health and Life Sciences, Brunel University London, Uxbridge UB8 3PH, UK; Juachi.Dimude@brunel.ac.uk (J.U.D.); Sarah.Smith@brunel.ac.uk (S.L.M.-S.); monja.st@gmx.com (M.S.)

**Keywords:** termination of DNA replication, fork collisions, RecG, homologous recombination, co-orientation of replication and transcription

## Abstract

Duplication of bacterial chromosomes is initiated via the assembly of two replication forks at a single defined origin. Forks proceed bi-directionally until they fuse in a specialised termination area opposite the origin. This area is flanked by polar replication fork pause sites that allow forks to enter but not to leave. The precise function of this replication fork trap has remained enigmatic, as no obvious phenotypes have been associated with its inactivation. However, the fork trap becomes a serious problem to cells if the second fork is stalled at an impediment, as replication cannot be completed, suggesting that a significant evolutionary advantage for maintaining this chromosomal arrangement must exist. Recently, we demonstrated that head-on fusion of replication forks can trigger over-replication of the chromosome. This over-replication is normally prevented by a number of proteins including RecG helicase and 3’ exonucleases. However, even in the absence of these proteins it can be safely contained within the replication fork trap, highlighting that multiple systems might be involved in coordinating replication fork fusions. Here, we discuss whether considering the problems associated with head-on replication fork fusion events helps us to better understand the important role of the replication fork trap in cellular metabolism.

## 1. Introduction

**Figure 1:**
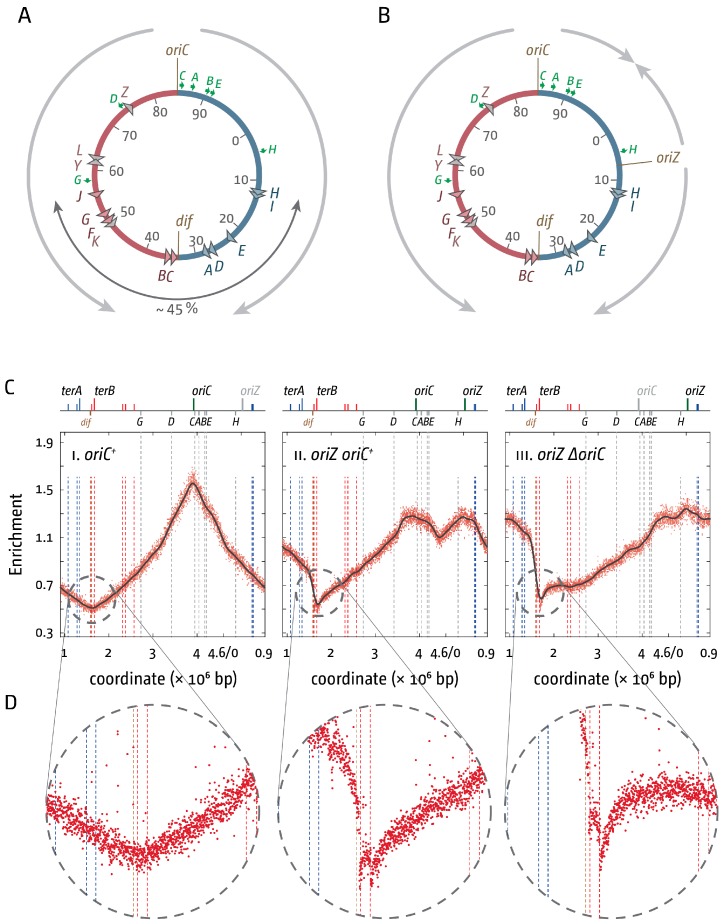
Replication fork fusion sites in *E. coli* cells with one and two replication origins. (**A**) Schematic representation of the replichore arrangement of the *E. coli* chromosomes. Direction of replication from the origins is indicated by grey arrows. The origin, *oriC*, and the *dif* chromosome dimer resolution sites are indicated. All *ter* sites are highlighted by triangles and identified by their corresponding letter (“A” indicates the *terA* site). The colour of the triangles corresponds with the replichore for which replication is permissive (*ter* sites highlighted in blue will block forks coming from the red replichore and vice versa). The secondary *ter* sites *terK*, *L* *Y*, and *Z* are shown in grey. Numbers represent the minutes of the standard genetic map (0–100 min). Green arrows represent location and direction of transcription of the 7 *rrn* operons *A–E*, *G*, and *H*; (**B**) Schematic representation of the replichore arrangement of the *E. coli* chromosome in cells with two replication origins. *oriZ* indicates the integration of a duplication of the *oriC* sequence near the *lacZYA* operon; (**C**) Marker frequency analysis of *E. coli oriC^+^*, *oriC^+^ oriZ*, and ∆*oriC oriZ* cells. The number of reads (normalised against reads for a stationary phase wild type control) is plotted against the chromosomal location. Note that the chromosomal location starts at 0.9 Mbp for a better visualisation of both replication origins. A schematic representation of the *E. coli* chromosome showing positions of *oriC* and *oriZ* (green line; grey if deleted/not present) and *ter* sites (above) as well as *dif* and *rrn* operons A–E, G, and H (below) is shown above the plotted data. The grey line shows a LOESS regression curve of the marker frequency data. Data are re-plotted from [10]; (**D**) Detail view of the native termination area of the *E. coli* chromosome. See text for details.

Chromosome replication in all cells studied is regulated by recruitment of the replication machinery to specific initiation sites (origins) where two forks are established and move in opposite directions until they meet either an opposing fork or the end of a chromosome. In *Escherichia coli*, replication initiates at *oriC* with the aid of the DnaA initiator protein. Once the DNA has been melted by DnaA, the leftward helicase is recruited first, followed by the rightward helicase [1]. Forks proceed with a speed of around 1000 nt/s [2], with the leftward fork being slightly ahead of the rightward fork [3]. Duplication of the chromosome is achieved when the two forks meet within a specialised termination zone opposite the origin. This area is flanked by ten primary polar *ter* sequences (*terA–J*) that are bound by the Tus terminator protein, which together act as replication fork pause sites. In combination, the *ter*/Tus complexes in the termination area form a replication fork trap that allows forks to enter but not to leave (Figure 1A) [4,5,6]. Thus, the chromosome is divided into two approximately equal halves or replichores, and 50% of the chromosome is replicated by the fork moving clockwise, while the other 50% is replicated by the fork moving counter-clockwise [7]. The termination area contains some specialised sequences, such as the *dif* site which, with the aid of the site-specific XerCD recombinase, is required to resolve chromosomal dimers that form as a consequence of an odd number of recombination events [8,9].

The replication fork trap dictates that the vast majority of head-on replication fork encounters take place within the boundaries of the termination area, and a scenario where one fork gets blocked at a *ter/*Tus complex might be an important part of replication termination (“fork trap model”). However, the precise role of the replication fork trap is not clear. Forks might naturally fuse in the termination area, with the fork trap mechanism only coming into action when one of the two forks is delayed by an obstacle such as a DNA lesion or a protein-DNA complex (“fork fusion model”).

Regardless of the precise mechanism, the fork trap mechanism poses a challenge to cells, because a fork blocked on its way from the origin will have to wait for a significant period before the second fork will be able to overcome the multiple *ter*/Tus complexes of the replication fork trap. A stalled fork will have to be restarted or the cell will be in danger of dying, explaining perhaps why replication restart pathways are very prominent in bacteria. Thus, the evolutionary advantage of having a fork trap mechanism in place must outweigh such disadvantages. A replication fork trap is not present in all bacterial species, demonstrating that it is not essential. On the other hand, the comparison of the components of the fork trap in the Gram-negative *E. coli* and the Gram-positive *Bacillus subtilis* have revealed no significant sequence or structural similarity, indicating that the fork trap systems might have evolved via convergent evolution [6], suggesting that in the organisms where it is present it has a very important physiological function. It is therefore a surprise that both in *B. subtilis* and *E. coli,* growth rate and cell morphology was reported to be indistinguishable from wild type cells if the replication fork trap was inactivated [11,12]. This observation questions our understanding of the precise function of the termination area.

Recently we published a series of results indicating that in *E. coli* the fusion of two replication forks can result in the formation of intermediates, which can trigger unwanted reactions such as substantial over-replication of the termination area and an increased number of recombination events. We have identified RecG helicase as well as 3’ exonucleases as key players for processing such intermediates, thereby preventing pathologies arising from fork fusion events [13,14,15,16,17,18]. Here we will discuss whether considering the problems associated with fork fusion events might help us to better understand the role that the termination area plays as part of cellular metabolism.

## 2. *ter*/Tus Complexes Block Replication Forks with High Efficiency

The existence of a specific termination system in *E. coli* was discovered by the observation that strains in which replication initiated in locations other than *oriC* still showed fork fusions opposite *oriC* [19,20,21], an observation that resulted in the identification of the innermost *ter* sites *terA*, *terB*, and *terC* [22,23,24] as well as the *termination utilization substance* (Tus protein) [25]. Further analysis revealed *ter* sites *terD*, *terE*, and *terF* [26,27,28,29]. The remaining primary *ter* sites *terG*, *terH*, *terI*, and *terJ* were revealed when the full sequence of the *E. coli* chromosome became available [30]. As shown in Figure 1A, these ten primary *ter* sites span roughly 45% of the entire *E. coli* chromosome. Four additional sites with weak fork pausing activity were also identified as *terK*, *terL*, *terY*, and *terZ* [31].

A *ter*/Tus complex blocks a replication fork in a polar manner [4,5,6,32,33,34]. However, *ter/*Tus complexes can be overcome, and they were initially described as replication fork pause sites [22,23], suggesting perhaps that the high number and the wide spread of *ter* sequences provides a fail-safe mechanism to keep replication forks within a certain region of the chromosome. It is therefore important to gauge how efficiently forks are blocked by *ter*/Tus complexes.

Bidnenko and co-workers demonstrated that replication forks were permanently blocked when reaching an ectopic *ter*/Tus complex [35]. To overcome the *ter*/Tus block, it was suggested that a second round of replication would run off the ends of the nascent strands of the stalled fork, leading to the generation of dsDNA ends that can engage in homologous recombination. This would then trigger recombination-driven replication restart [35,36,37]. Thus, while forks blocked at a *ter*/Tus complex can be restarted eventually, a single replisome will be stably arrested at a *ter*/Tus complex for a significant period of time, as recently demonstrated [38], with RecA being important for maintaining stability at forks [39]. In line with this idea, it was shown that the inversion of one *ter* site, which blocked replication of ~1 kb of the chromosome in the presence of Tus protein, caused severe filamentation [40]. Additional support comes from the observation that the innermost *ter* sites *terB*, *terC*, *terA*, and *terD* have a high fork pausing efficiency [31,41]. In vivo paused forks were only detectable at those four locations [31].

Recently we conducted marker frequency analyses (MFA) by deep sequencing to establish replication profiles [18,42,43] in cells in which a second copy of the *oriC* sequence, termed *oriZ*, was integrated roughly half-way into the right-hand replichore [10,18,44]. Such a strain is expected to show an asymmetric replication profile, as synthesis initiating at *oriZ* traversing clockwise will only duplicate 25% of the chromosome before reaching the replication fork trap, while replication initiating at the original *oriC* and proceeding counter-clockwise will still have to duplicate 50% of the chromosome (Figure 1B). In cells in which *oriC* is deleted, this asymmetry will be even more pronounced, as the fork travelling counter-clockwise will have to duplicate 75% of the entire chromosome.

Our data confirmed the predicted asymmetry of the replication profile (Figure 1C). Because forks coming from *oriZ* are able to reach the replication fork trap much earlier than forks coming from *oriC* and are blocked at *terC*/Tus or *terB*/Tus, on a population basis there will be significantly more cells that have replicated the area between *terA* and *terC/B* than the other side, resulting in a noticeable step of the MFA profile. The relevant section of the termination area (Figure 1D) shows two specific low points that coincide well with *terC* and *terB*. Given that two defined stops are visible in the profile, our data support the idea that a single *ter*/Tus complex is not 100% efficient in blocking approaching replication forks. However, there is little indication of synthesis proceeding beyond *terB*, demonstrating that *terC* and *terB* together provide a strong block to replication [10]. This was confirmed further by the replication profile of *ΔoriC oriZ* cells, which is even more asymmetric (Figure 1C (iii)). However, forks still terminate at either *terC* or *terB* (Figure 1D), and only a fraction of forks progress into the opposite replichore [10].

Our experiments also confirmed that the replication fork trap provides a serious problem to cells if progression of one of the forks is delayed by obstacles such as DNA lesions or protein-DNA complexes. We showed that the doubling time of *ΔoriC oriZ* cells in LB broth was, at 40 min or longer, twice as long as in wild type cells, and suppressor mutations rapidly accumulated, demonstrating that the initiation of synthesis from an ectopic location causes severe problems for cells [10]. It was shown before both in *E. coli* and *B. subtilis* that the most severe problems for ongoing replication arise at the highly transcribed *rrn* operons, which slow or block progressing replication forks, and it was shown that RecBCD processing of dsDNA ends is required to allow replication to restart [45,46,47,48]. In line with these observations we observed that the replication profile of *ΔoriC oriZ* cells shows some deviations in comparison to the profile observed in wild type cells, with the two most noticeable being located around 4.2 Mbp and 0.23 Mbp (Figure 1C) [10]. These deviations coincide with the location of the *rrn* operons *H* (0.229 Mbp) and the *CABE* cluster (3.94–4.21 Mbp). Replication coming from *oriZ* will progress into these areas in the wrong orientation, which triggers head-on collisions between replication and transcription. These collisions may slow down overall progression of replication forks, which would result in a steeper gradient of the replication profile in this area when compared to the wild type profile, providing an explanation for the “steps” in the replication profile.

**Figure 2:**
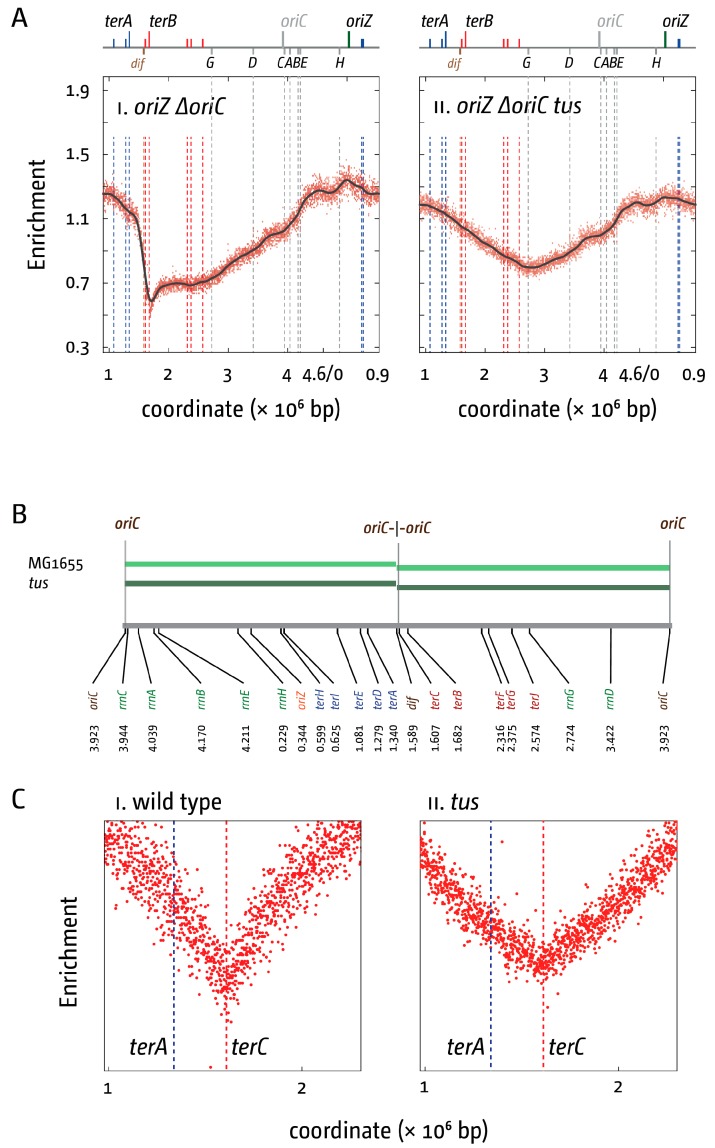
Replication termination in *E. coli* cells in the presence and absence of a functional replication fork trap. (**A**) Marker frequency analysis of *E. coli* ∆*oriC oriZ* cells in which the replication fork trap in the termination area was inactivated by deletion of the *tus* gene. The number of reads (normalised against reads for a stationary phase wild type control) is plotted against the chromosomal location, starting at 0.9 Mbp for a better visualisation of both replication origins. A schematic representation of the *E. coli* chromosome showing positions of *oriC* and *oriZ* (green line; grey if deleted/not present) and *ter* sites (above) as well as *dif* and *rrn* operons A–E, G, and H (below) is shown above the plotted data. The grey line represents a LOESS regression curve. Data were re-plotted from [10]; (**B**) Replichore parameters of *E. coli* cells in the presence and absence of a functional replication fork trap. A schematic of the *E. coli* chromosome is shown at the bottom, highlighting the location and coordinates of *oriC*, the chromosome dimer resolution site *dif*, *ter* sites *A–J*, and *rrn* operons *A–E*, *G*, and *H*. The relevant genotypes are stated on the left, with coloured bars representing the length of the replichore from each of the origins present, as calculated by the LOESS minima. The arithmetic mid-point between the origin is highlighted. The LOESS minima were determined from data sets published in [18]; (**C**) Detail view of the fork fusion area of the *E. coli* chromosome in the presence and absence of a functional replication fork trap. Data were re-plotted from [18].

An rpoB*35 point mutation, which destabilises ternary RNA polymerase complexes [49,50], not only suppresses the long delay in the doubling time but also reduces the deviations of the replication profile at all *rrn* operons in a ΔoriC oriZ rpo* background [10], supporting the idea that head-on replication-transcription conflicts are responsible for the delay observed. In addition, our data confirm that forks trapped in the terminus area pose a problem to cell cycle progression and viability if the other fork is slowed or blocked by any given obstacle, as inactivation of the Tus terminator protein robustly suppressed the long delay in cell division of *ΔoriC oriZ* cells, leading to a rather dramatic change of the replication profile (Figure 2). The “step” in the termination area of *ΔoriC oriZ* cells disappeared in the *tus* derivative, resulting in an almost perfectly symmetrical replication profile opposite *oriZ* [10]. Given the observed problems to cell growth and division caused by the replication fork trap, there must be a distinct evolutionary advantage for maintaining this specific chromosomal arrangement.

## 3. The Location of Fork Fusion Events

In *E. coli* MG1655 the location of *oriC* is at 3.923 Mbp, which, with a genome size of 4,639,675 bp, puts the theoretical mid-point opposite *oriC* at 1.603 Mbp in close proximity to *terC* (located at 1.607 Mbp). Even if the fork duplicating the left-hand replichore is slightly ahead of the fork replicating the right-hand replichore [3], forks will fuse closer to *terC* than to *terA* (located at 1.340 Mbp) if they travel with comparable speed. In line with this idea, Duggin and Bell found via 2D DNA gel electrophoresis that the strongest signal for blocked forks was at *terC*, followed by *terA* and *terB* [31]; a result reproduced in other labs [51]. Given that forks stalled at *ter*/Tus complexes can be detected in vivo, progression of the two forks must differ enough on a regular basis to force one of the forks to be blocked by the replication fork trap.

However, it is not very easy to evaluate how often forks fuse with one fork arrested by a *ter/*Tus complex. By using the available sequence information it was observed that the change in the GC skew points towards the *dif* chromosome dimer resolution site as the main fork fusion site, rather than any of the *ter* sites [52,53,54,55]. Differences in the types and rates of single base mutations in the leading and the lagging strand are thought to result in asymmetric replication-related mutation pressures, leading to the accumulation of G over C in the leading strand [55,56,57]. By using octamer sequences that were specifically skewed to the leading strand, it was shown that the distribution of these octamers switched from one strand to another near *dif*, leading to the suggestion that replication forks might be halted by *dif* under normal conditions [54]. In a computer modelling study, Kono and colleagues tried to investigate this question further [58]. They analysed three different scenarios: forks freely fusing within the innermost *ter* sites, termination with one fork arrested at a *ter/*Tus complex, and forks being halted at *dif*. The work presented suggests that for the simulation parameters used, forks being halted by *dif* produced a GC skew pattern that provided the worst fit observed. Both forks fusing freely and forks fusing at *ter/*Tus complexes produced significantly better results [58].

We used our high-resolution replication profiles to address whether forks fuse freely in between the innermost *ter* sites or whether one fork regularly gets blocked at a *ter/*Tus complex. If replication by one fork is faster under normal growth conditions, causing it to reach a *ter*/Tus complex before the other fork arrives, then it should be expected that in a *Δtus* background the fork normally blocked will be able to proceed into the opposite replichore, which should shift the point where forks fuse. Thus, by analysing LOESS regression curves for the replication profiles of wild type and *Δtus* cells we compared whether there is any change in the position of the lowest point of the replication profile [10,18]. We found that the LOESS minimum both in wild type and *tus* cells was located precisely at the same location at 1.591 Mbp, which is in between *dif* (1.589 Mbp) and *terC* (1.607) (Figure 2B,C) [10,18]. This suggests that on a population level, the majority of fork fusion events are taking place within the innermost *ter* sites regardless of whether the replication fork trap is active or inactive. However, we did notice that the shape of the fork fusion zone changes (Figure 2C). The “valley” was less defined in the absence of Tus, suggesting more variability in the precise fork fusion location [18], in line with the idea that the fork fusion point is influenced by a number of different parameters, including forks being blocked at transcribing RNA polymerase complexes, tightly bound protein-DNA complexes, or DNA lesions. This is supported by our results in *oriC^+^ oriZ tus* cells. Introduction of an additional replication origin leads to a shift of the area where forks are fusing. Upon inactivation of Tus, forks will freely move until they fuse. Our data show that in this chromosomal set-up, a fork fusion area is observed that is still well-defined (Figure 2A) [10], in line with the idea that on a population level forks move with comparable speeds.

Interestingly, Kono and colleagues proposed that the position of the *dif* site might be co-evolving with the main location of where forks normally fuse [58]. In cells without Tus protein the main location of fork fusion events is not changed (Figure 2B,C) [10,18], suggesting that the same might be true for the replication fork trap; it might be located around the area where replication forks naturally fuse, rather than enforcing the location of fork collisions.

## 4. Coordinating Replication and Transcription

Regardless of what defines its precise location, the data in the previous section suggest that the replication fork trap will efficiently prevent any fork from leaving the termination area. This enforces a strong directionality of replication, with each replichore always being replicated in a defined orientation. This directionality might provide an advantage, and it was suggested that co-orientation of replication and transcription might be an important contributing factor [59,60,61]. Both processes use the same template strand, but transcription moves at a pace 10–20 times slower than replication [62,63], making conflicts unavoidable. Highly transcribed genes are preferentially located on the leading strand template in many bacterial species, which allows replication and transcription to move co-directionally [59,61,64,65]. While in *E. coli* the overall co-orientation is only 54%, 93% of highly transcribed genes that code for ribosomal proteins show co-directionality of replication and transcription [59,65]. In other bacteria, the general co-orientation is even higher (>70% overall in species such as *Bacillus subtilis* and *Mycoplasma pneumoniae*), with an even more pronounced co-directionality of genes that code for ribosomal proteins [65]. In contrast, the analysis of replication and transcription directionality in eukaryotic cells has revealed no overall bias, suggesting, effectively, a random orientation of open reading frames [66]. However, replication-transcription encounters must cause some problems in eukaryotic cells, as well as in yeast, a replication barrier was found which prevents forks from entering the highly transcribed ribosomal DNA repeats in a head-on orientation [64,67,68].

The co-directional movement of replication forks and transcribing RNA polymerase complexes, particularly of highly transcribed genes, implies that head-on encounters of replisomes with RNA polymerase complexes might be rather problematic [69,70,71,72], a result readily supported by the problems demonstrated to occur both in *E. coli* and *B. subtilis* at *rrn* operons replicated in the wrong orientation [45,46,47,48,73], as well as in our own work in *E. coli* *ΔoriC oriZ* cells in which distinct distortions of the replication profiles were observed at the locations of the *rrn* operons *H* (0.229 Mbp) and the *CABE* cluster (3.94–4.21 Mbp) [10]. In addition, we observed very similar deviations in other backgrounds in which *rrn* operons are replicated opposite to normal. In cells lacking RNase HI, origin-independent DNA synthesis can be initiated at DNA:RNA hybrids [74], leading to a small number of reasonably well-defined locations where replication is initiated [13,51,75]. One of these initiation sites is located such that replication progresses into the *rrnCABE* operon cluster in the wrong orientation if firing of *oriC* is inhibited by a temperature-sensitive *dnaA46* allele. In this background, similar deviations of the replication profile are observed at the location of the *rrnCABE* operon cluster, which again can be partially suppressed by an rpo* point mutation [13], supporting the idea that head-on collisions between replication and transcription slows replication significantly [10,13].

An additional strong supporting argument for this hypothesis comes from the identification of a suppressor mutation that allowed fast growth of *∆oriC oriZ* cells. Wang and colleagues described the construction of both *oriC^+^ oriZ*, and *∆oriC oriZ* cells and reported a very similar doubling time for both [44]. As our own results indicated very clearly that *∆oriC oriZ* show a significant growth defect which can be suppressed by both rpo* and *tus* mutations [10] (see above), we suspected that the *∆oriC oriZ* construct described [44] might have acquired a suppressor mutation. We used whole genome sequencing data to identify the nature of this suppressor mutation. Intriguingly, the replication profile obtained provided a stunningly simple explanation for the reported short doubling time. In the fast growing strain, a gross chromosomal rearrangement was found, which inverts the chromosome section from the deleted *oriC* region (3.920 Mbp) to the *leuABC* area (0.082 Mbp). Thus, while the region between *leuABC* and *oriZ* (0.334 Mbp), including the *rrnH* operon, remains in its original orientation, the section between *leuABC and ΔoriC* is inverted, which includes the entire *rrnCABE* operon cluster. Thus, the inversion of this 800 kb stretch in cells replicating from *oriZ* will re-align the directionality of replication and transcription and any issues arising from head-on replication-transcription conflicts in this area are simply eliminated [10]. The fact that this gross chromosomal rearrangement arose so readily as a spontaneous suppressor mutation strongly reinforces the idea that head-on collisions between replication and transcription have a severe impact on ongoing chromosomal replication.

**Figure 3:**
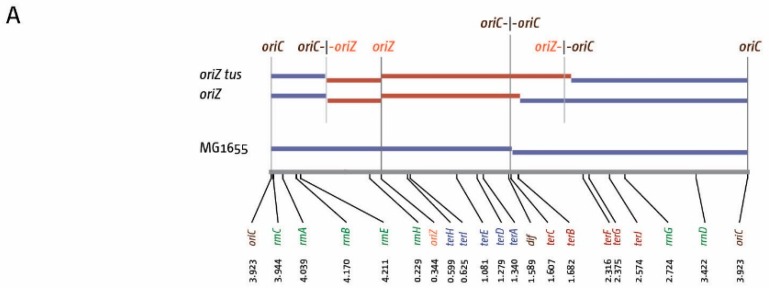

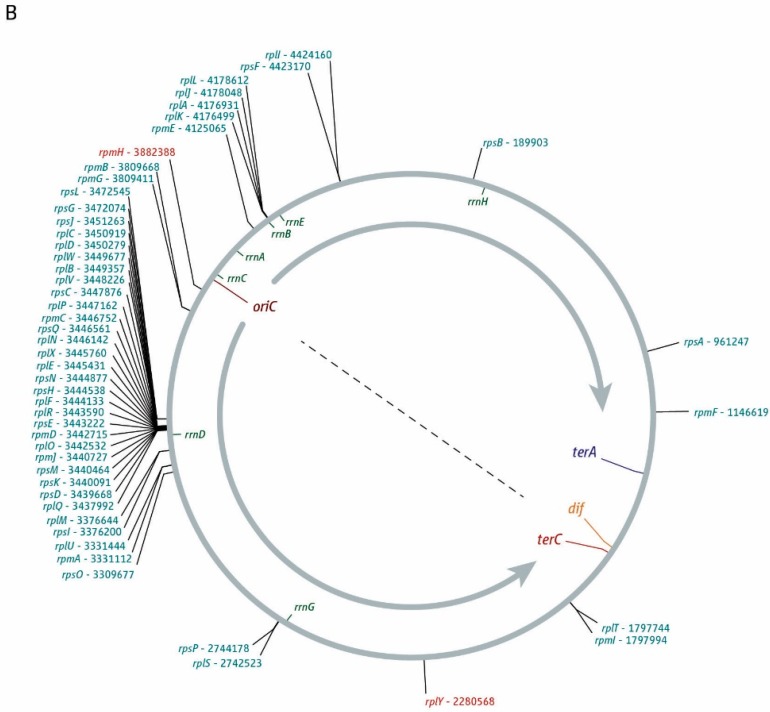
Replication and transcription parameters of *E. coli* cells with one and two replication origins. (**A**) Replichore lengths in *E. coli* cells with two replication origins in the presence and absence of a functional replication fork trap. A schematic of the *E. coli* chromosome is shown at the bottom, highlighting the location and coordinates of *oriC*, *oriZ*, the chromosome dimer resolution site *dif*, *ter* sites *A–J*, and *rrn* operons *A–E*, *G*, and *H*. The relevant genotypes are stated on the left, with coloured bars representing the length of the replichore from each of the origins present, as calculated by the LOESS minima. Arithmetic mid-points between origin(s) are highlighted by grey lines. Data were re-plotted from [10]; (**B**) Location and orientation of *rrn* operons as well as genes coding for ribosomal proteins within the *E. coli* chromosome. *oriC*, the innermost *ter* sites and the *dif* chromosome resolution site are indicated. Orientation of replication within a replichore is indicated by a grey arrow. Locations of *rrn* operons *A–E*, *G*, and *H* are shown on the inside. All *rrn* operons are transcribed co-directionally with DNA replication (green). Locations of genes encoding ribosomal proteins are indicated on the outside. Genes transcribed co-directionally with replication are shown in cyan, genes transcribed in head-on orientation relative to replication in the replichore are shown in red.

Given that head-on conflicts between replication and transcription pose a significant threat to cells, the hypothesis that the replication fork trap prevents one fork entering the opposite replichore in the wrong orientation if the second fork is delayed at an obstacle seems tempting. However, a prediction of this theory would be that forks escaping the termination area would be slowed down, as they would suffer from an increased number of head-on collisions when entering the wrong replichore. We were able to investigate this question in *oriC^+^ oriZ tus* cells. In a background in which the replication fork trap is inactivated, forks coming from *oriZ* in the clockwise orientation will be able to proceed past *terC/B* into the opposite replichore. If they slow down relative to the fork moving counter-clockwise coming from *oriC*, the termination point of the replication profile should be shifted away from the arithmetic mid-point between *oriC* and *oriZ*, located at 2.1335 Mbp, towards the termination area. However, we were surprised to find the opposite: the LOESS minimum was at 2.199 Mbp, over 60 kb in the direction of *oriC* (Figure 3A) [10]. While we do not have any direct indication about the speed of individual replication forks, this result suggests that the fork coming from *oriC* and going in the native direction must be, overall, slower than the fork coming from *oriZ* replicating in the wrong orientation from the termination area onwards.

The strongest deviations of the replication profiles were consistently observed at the *rrn* operons [10,13,46,47,48]. However, all *rrn* operons are relatively close to *oriC*. For DNA synthesis escaping the termination area to even reach *rrnG*, the most origin-distal *rrn* operon, forks would have to replicate more than 1 Mbp (Figure 1), which would be a rare event. But *rrn* operons are not the only highly-transcribed genes. We therefore analysed the location and orientation of a number of genes that will be highly transcribed under fast growth conditions. The orientation of transcription of all genes encoding for ribosomal proteins (Figure 3B) is highly biased towards being co-directional with replication (100% in the right-hand replichore and 95% in the left-hand replichore). However, similarly to *rrn* operons, the majority of these genes are located close to *oriC*, with 62% forming a tight cluster located between 3.31 and 3.47 Mbp of the chromosome, relatively close to *oriC*, while only 15% are located in the terminus half of the chromosome.

**Figure 4:**
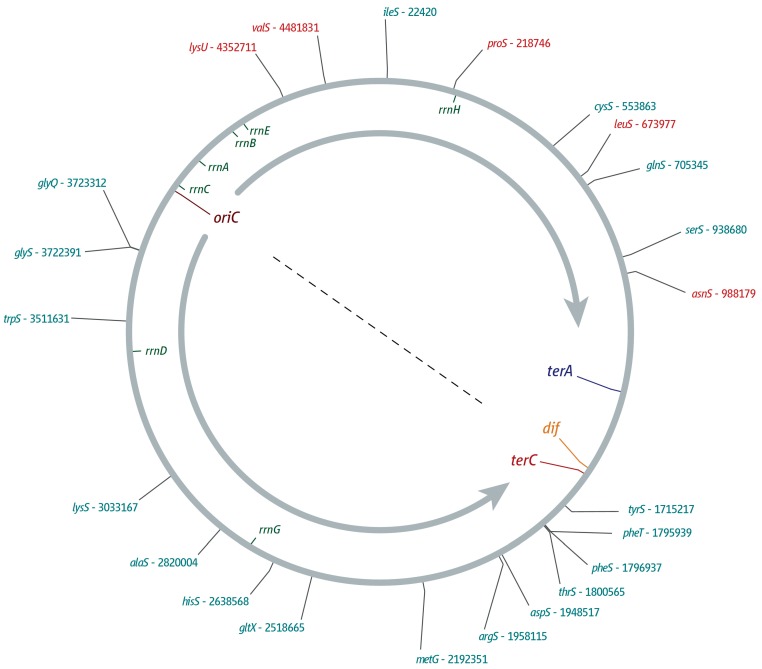
Location and orientation of genes coding for aminocyl tRNA synthetases within the *E. coli* chromosome. *oriC*, the innermost *ter* sites, and the *dif* chromosome resolution site as well as locations of *rrn* operons *A–E*, *G*, and *H* are indicated. Orientation of replication within a replichore is indicated by a grey arrow. Locations of genes encoding aminoacyl tRNA synthetases are indicated on the outside. Genes transcribed co-directionally with replication are shown in cyan, genes transcribed in head-on orientation relative to replication in the replichore are shown in red.

Aminoacyl-tRNA-synthetase genes (Figure 4) are much more evenly distributed and co-direc­tionality is less pronounced. In the left-hand replichore, 85% of genes are co-oriented (12 from 14), but only 45% are co-oriented in the right-hand replichore (4 from 9).

**Figure 5:**
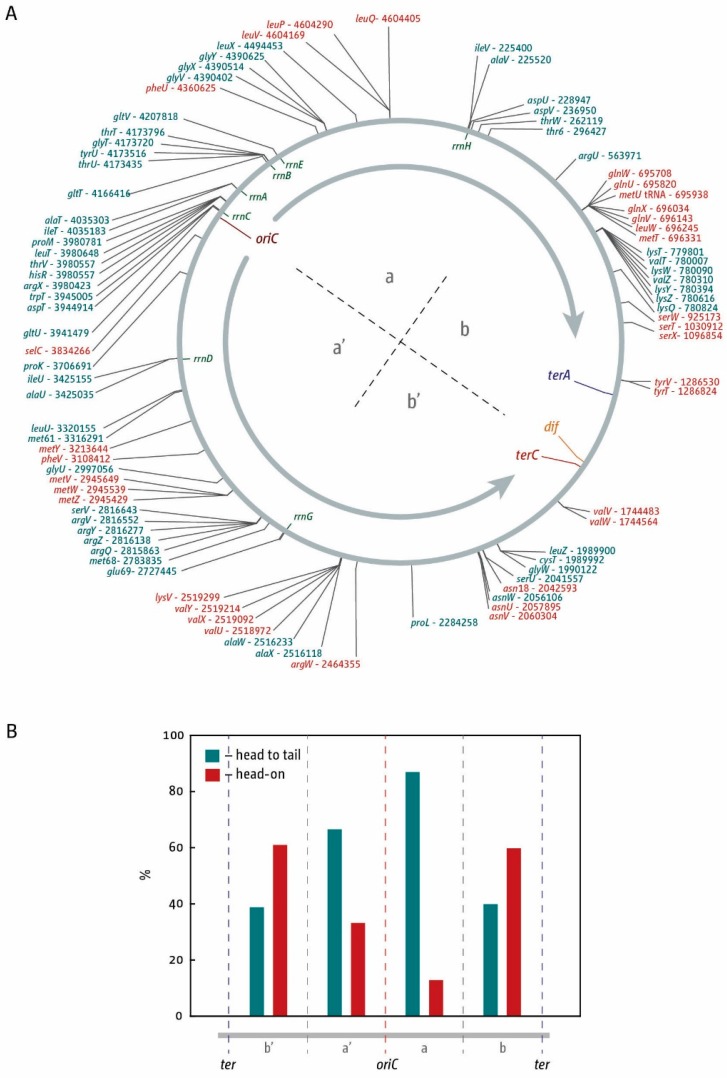
(**A**) Location and orientation of genes coding for tRNAs within the *E. coli* chromosome. *oriC*, the innermost *ter* sites, and the *dif* chromosome resolution dimer site as well as locations of *rrn* operons *A–E*, *G*, and *H* are indicated. Orientation of replication within a replichore is indicated by a grey arrow. Locations of tRNA genes are shown on the outside. Genes transcribed co-directionally with replication are shown in cyan, genes transcribed in head-on orientation relative to replication in the replichore are shown in red; (**B**) Frequency of head-on (red) and head to tail (cyan) orientation relative to DNA replication of tRNA genes per chromosomal quarters. Quarters a and a’ are origin-proximal, as shown in (A), while b and b’ are origin-distal.

The distribution of tRNA genes revealed unexpected features (Figure 5A). While the overall distribution of genes is relatively even, we found that the majority of origin-proximal genes are co-oriented with replication. However, genes located in the origin-distal half of the chromosome are preferentially oriented in the head-on orientation (Figure 5A,B), with genes in head-on orientation not being restricted to rare codons. This suggests that replication escaping the termination area might in fact encounter fewer problems than replication coming from *oriC*.

In addition, high levels of transcription were shown to interfere with replication if both processes are co-oriented, even though the impact is not as severe as in head-on orientation [72,76]. Thus, the cluster of the ribosomal protein genes and the *rrn* operons *D* and *G* might contribute towards slowing forks coming from *oriC*. Both observations together might go some way towards explaining why forks escaping the termination area are able to progress more quickly than forks coming from *oriC* [10].

Our current experimental data only cover the area outside of *terC/terB* and fork progression is only determined as the speed of the two replisomes relative to each other. However, taken together there is very little direct evidence that the main purpose of the termination area is to prevent head-on collisions between replication and transcription. Genes transcribed at very high levels are relatively distant from the fork trap area, forks escaping the termination area progress with little indication of problems, and, in addition, preventing one replisome from entering the opposite replichore does not explain why in *E. coli* the replication fork trap covers almost 50% of the chromosome. Two *ter*/Tus complexes in relative proximity are fully sufficient to hold fork progression rather efficiently, suggesting that there is little need for three additional backup sites, which, in addition, are relatively far away. We certainly cannot exclude that the prevention of head-on encounters is a welcome side effect of having the replication fork trap, but the question remains whether there might be a more important reason for maintaining a replication fork trap.

## 5. The Replication Fork Trap Contains Over-Replication

One of the reasons that the precise function of the replication fork trap is hard to define is the fact that it can be inactivated with little consequence [5,11,12] and, in addition, that not all bacterial species possess one in the first place. What phenotypes are associated with its inactivation? Horiuchi and co-workers were able to show that *ter* sequences can act as recombination hot-spots in the presence of Tus protein in *E. coli* [77,78]. This might be explained by recombination triggered at forks blocked at *ter*/Tus once they are reached by a second round of synthesis [35,36,37]. In addition, we will discuss an alternative trigger of recombination later in this section.

Furthermore, it was reported for a plasmid-based in vitro replication system that *ter* sites which prevent the direct fusion of replication forks prevent over-replication of plasmid DNA in the presence of Tus protein [68,79]. Similarly, over-replication was also reported for in vivo experiments in systems lacking Tus. Krabbe and co-workers [80] investigated replication intermediates of the R1 plasmid. Replication is established at *oriR* and a single processive fork moves uni-directionally until it reaches a *ter*/Tus complex in close proximity to *oriR* [81]. It was observed that in the absence of a functional *ter*/Tus complex, maintenance of the R1 plasmid became unstable and a variety of complex DNA structures were accumulating, including complex branched structures, multimeric forms and rolling circle replication of the R1 plasmid [80]. The authors suggested that the helicase of the replication fork reaching the already replicated area might displace the existing nascent ends, thereby generating intermediates which allow the continuation of replication [80].

The observed over-replication is unlikely to be a peculiarity of the used plasmids, as it was also observed in the chromosomal termination area. Markovitz observed low levels of chromosomal over-replication in the absence of Tus protein in *E. coli*, an effect that was significantly stronger if additional mutations were added, such as point mutations in the *polA* gene, suggesting that DNA polymerase I might have a role in bringing termination of DNA replication to a successful conclusion [82]. Similarly, the phenotypes associated with the absence of the replication terminator protein RTP in *B. subtilis* suggest problems with terminating DNA replication accurately, resulting in effects such as an increase of chromosome dimers [83].

**Figure 6:**
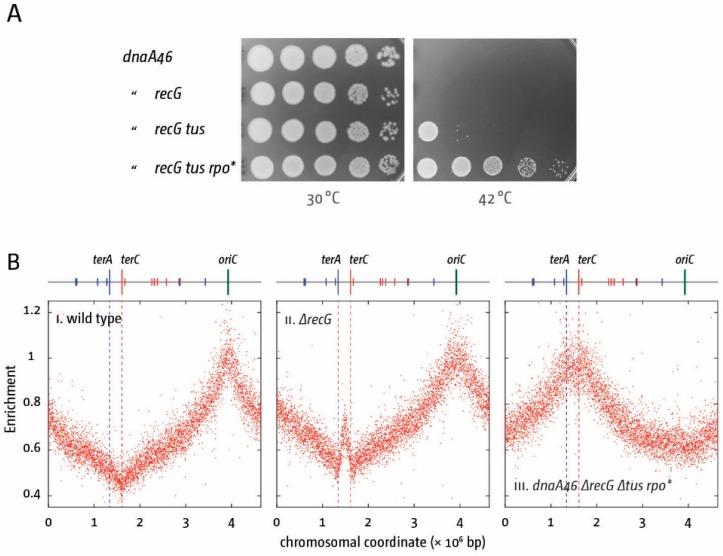
DnaA-independent replication triggered by the absence of RecG can contribute to cell growth if the replication fork trap is inactivated. (**A**) Spot dilution assay showing the effect of *recG*, *tus* and *rpoB* mutations on growth without DnaA (*dnaA46* at 42 °C); (**B**) Marker frequency analysis of *E. coli* cells in exponential phase. The number of reads (normalised against the reads for a stationary wild type control) is plotted against the chromosomal location. Positions of *oriC* (green line) and primary *ter* sites are shown above the plotted data with red and blue lines representing the left and right replichore as depicted in Figure 1A. Data were re-plotted from [18].

While clearly resulting in defined phenotypes, the observed defects relating to the absence of the replication fork trap system are all very mild under laboratory growth conditions. However, recently we were able to identify a phenotype for Δ*tus* in cells lacking RecG helicase that was more pronounced (Figure 6A) [13,18]. Kogoma and co-workers discovered that inactivation of RecG in *E. coli* enables initiation of synthesis independent of the normal *oriC* [74,84]. The analysis of replication profiles in cells lacking RecG demonstrated that the synthesis arising is restricted to the termination area (Figure 6B) [18], a result confirmed in other labs [85,86]. We speculated that inactivation of the replication fork trap might allow forks to progress, enabling growth of *recG* cells in the absence of *oriC* activity. This was indeed observed. We found that *dnaA(ts) recG* *tus* cells show growth at restrictive temperature [18], one of the first easily demonstrable phenotypes of a Δ*tus* deletion (Figure 6A). However, replication coming from the termination area will replicate the chromosome in the wrong orientation, leading to conflicts between replication and transcription especially at highly transcribed loci such as *rrn* operons. Partial alleviation of these conflicts by introduction of an rpo* point mutation resulted in strains which can grow robustly even if DnaA is inactivated (Figure 6A) or the entire *oriC* area is deleted [13,18]. Replication profiles of a dnaA(ts) recG tus rpo* background grown at restrictive temperature revealed an effectively inverted replication profile (Figure 6B). There is no indication of initiation of synthesis at *oriC*. Instead, a broad peak of synthesis is observed in the terminus half of the chromosome [18].

What is causing the observed over-replication in *recG* cells? It certainly is not as simple as a defined activity of a cryptic origin normally suppressed by RecG, as initiation of synthesis can be substantially modulated. Linearisation of the *E. coli* chromosome significantly reduces the over-replication observed in *recG* cells. If the chromosome is linearised in the termination area, forks coming from *oriC* will not fuse any more, but will run into a chromosome end. Replicating such an end comes with its own challenges, explaining why a low level of over-replication remains detectable as long as *oriC* is active [18]. However, over-replication is much reduced and completely abolished in *dnaA(ts) recG* cells with a linearised chromosome following a shift to restrictive temperature [18]. Thus, a circular chromosome is a prerequisite for the over-replication to occur, while the remaining low level of synthesis depends entirely on forks coming from *oriC*. In addition, the over-replication is dramatically increased in *oriC^+^ oriZ* strains lacking RecG [18]. It is not easily explained how integration of *oriZ* some 1 Mbp away should massively increase the activity of this cryptic origin in the termination area. It seems far more likely that perhaps the increased number of forks blocked at *ter*/Tus complexes in *oriC^+^ oriZ* cells might contribute towards the over-replication. However, it is important to note that the over-replication observed in cells lacking RecG is not dependent on *ter*/Tus complexes, as recG tus rpo* cells can grow robustly even if *oriC* is deleted, despite the absence of Tus (Figure 6A) [18].

**Figure 7:**
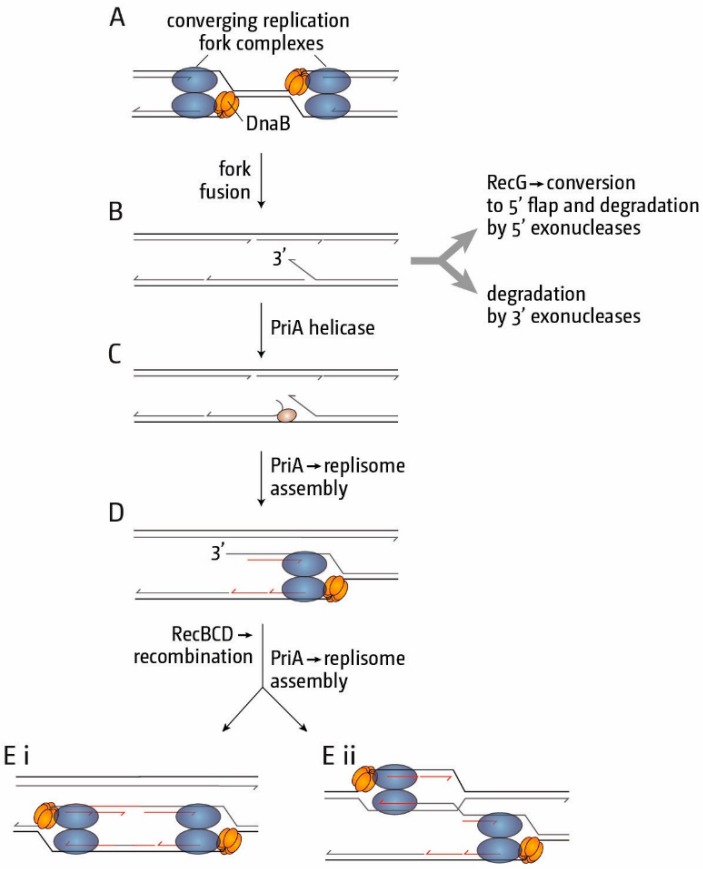
Schematic illustrating how replication fork fusions might lead to the formation of new divergent forks via PriA-mediated replisome assembly and RecBCD-mediated recombination, and how this can be normally suppressed by RecG and/or 3’ exonucleases. The formation of a 3’ flap can occur at both forks. However, for simplicity the schematic details only one such reaction. See text for further details.

What might trigger the over-replication observed? The fusion of replication forks is unique to the termination area, and fork fusion events might generate intermediates which result in the recruitment of additional replication forks if they are not processed by RecG, explaining why chromosome linearisation efficiently suppresses the over-replication, as head-on fork fusions are eliminated. A clue about one of the molecular intermediates came from the observation that synthesis in the termination area in *recG* cells is strictly dependent on PriA helicase activity [18]. Even more specifically, it is entirely dependent on the ability of PriA helicase to process 3’ flap structures [18]. The *srgA1* allele of *priA* encodes a mutant protein (PriA L557P) which unwinds a replication fork with both a leading and a lagging strand at the branch point as efficiently as wild type PriA, but it has lost the ability to unwind a fork in which the leading strand is missing [87] the equivalent of a 3’ flap. This led us to suggest that a 3’ flap structure persists in the absence of RecG [18], an idea supported by the observation that cells lacking 3’ exonucleases also showed over-replication in the termination area [18,86]. Such 3’ flap structures could be generated if the replicative helicase of one fork, which encircles the lagging strand template [88], would displace the nascent leading strand of the opposing fork (Figure 7A,B), similar to the displacement of nascent strands suggested for the observed over-replication of R1 plasmid [80]. Because displacement of a 3’ flap is not observed with DnaB alone [89], it was suggested that nascent strand displacement is a particular risk following collision between fully-fledged replisomes [15,18], an idea supported by in vivo observations [80,82]. In wild type cells 3’ flaps can be eliminated by 3’ ssDNA exonucleases, but they are also excellent substrates for RecG [90,91,92] which, via unwinding, will convert them to 5’ flaps that could subsequently be removed by 5’ ssDNA exonucleases (Figure 7B) [13,16,17,18,93].

In the absence of either RecG or 3’ exonucleases, a 3’ ssDNA flap will persist for longer and will provide a substrate that PriA could exploit to trigger assembly of a new replication fork (Figure 7C). The affinity of RecG for 3’ flaps is 10-fold higher than that of PriA [92], which explains why over-replication in the presence of RecG (and 3’ exonucleases) is very low [82]. However, if PriA establishes a new fork at a 3’ flap structure, progression of this fork would generate a duplex arm with a free DNA end (Figure 7D), which may then invade the re-replicated DNA behind the fork (or the sister duplex) via the action of RecBCD and RecA recombinases (Figure 7E (i,ii)) [13,14,15,16,17,18]. This in turn would establish a D-loop that could also be targeted by PriA to establish yet another fork moving in the opposite direction. Indeed, we demonstrated that the over-replication observed in *recG* cells depends on the recombinase activity of RecBCD, and growth of dnaA(ts) recG tus rpo* cells is prevented by the deletion of recB [18], which eliminates the dsDNA exonuclease activity of RecBCD while leaving the recombinase activity intact [94], but not recD [18], which eliminates the exonuclease activity [94]. The newly established forks would be blocked by the *ter*/Tus traps as they proceed towards *oriC*, explaining why over-replication is tightly restricted to the termination area. The idea of accumulating 3’ flaps is also consistent with the inviability of *recG* cells lacking three exonucleases each capable of removing a 3’ flap, and the restoration of viability when the helicase activity of PriA required for the observed over-replication is eliminated [17,18].

**Figure 8:**
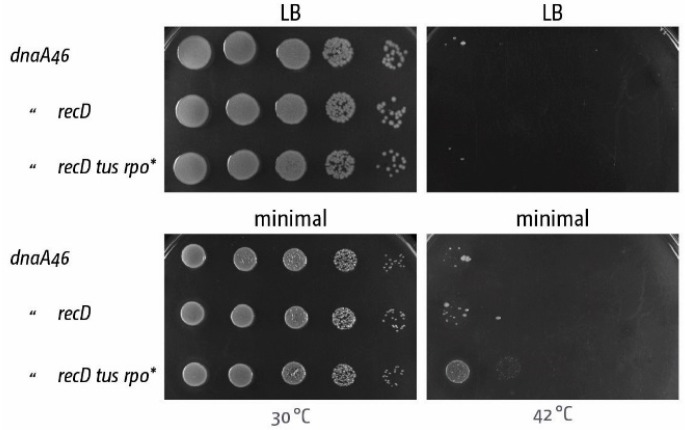
DnaA-independent growth in cells triggered by the absence of RecD contributes very little to cell growth. Spot dilution assay demonstrating that *recD* contributes little to growth without DnaA (*dnaA46* at 42°C) even in the presence of *tus* and rpo* mutations (compare with the effect observed in the absence of RecG in Figure 6A).

The scenario described provides an additional explanation why the termination area might be a recombination hotspot [77], as both 3’ ssDNA ends and dsDNA ends are substrates for recombination. It identifies both RecG and 3’ exonucleases as key factors for processing fork fusion intermediates and it is easy to imagine roles for other proteins, such as RecBCD or DNA polymerase I, at the final stages of DNA replication [82,86]. The over-replication observed in a *recD* mutant, which inactivates the RecBCD exonuclease activity, was suggested to be caused by forks transiently passing each other, resulting in over-replicated areas which are then degraded by RecBCD exonuclease activity [86]. However, the amount of over-replication triggered is very mild in comparison to the levels seen in *recG* cells. When we crossed a *recD* allele into a dnaA(ts) tus rpo* background and investigated growth at restrictive temperature, we found that no growth can be observed in rich medium, and only very limited growth is observed on minimal medium (Figure 8; cf with Figure 6).

If fork fusions can lead to over-replication, the origin-independent synthesis should be observed in an *oriC^+^ oriZ recG* background in the secondary termination site located between *oriC* and *oriZ*. Our data suggest that this is indeed the case [18]. However, the situation is complex, because the defined peak of synthesis observed in *recG* single mutants is much reduced in a *recG tus* double mutant. Instead, *recG tus* cells show a much more rounded “valley” of the termination zone [18]. This is consistent with forks being able to progress beyond the boundaries of *terC* and *terA*, which increases over-replication in a much wider area in the terminus half of the chromosome, rather than resulting in a defined peak. Thus, as no replication fork trap is present in the ectopic termination area in *oriC^+^ oriZ recG* cells, we would expect to see a similar mild increase of the marker frequency in the general area where forks fuse, which is exactly what we observed [18]. However, it remains to be established whether reconstitution of a replication fork trap in the ectopic termination area results in the formation of a sharp peak of over-replication in the absence of RecG, as observed in the native termination area.

Both RNase HI and RecG can process DNA:RNA hybrids [95,96,97,98], and it was suggested that origin-independent synthesis observed both in *rnhA* and *recG* cells is initiated at R-loops. Indeed, a common basis for initiation might account for the fact that cells lacking RecG and RNase HI both show a peak of synthesis in the termination area of the chromosome [99], even though other initiation sites are observed in the absence of RNase HI which are missing in cells lacking RecG [13,18,51,75]. However, there is no indication that the targeting of 3’ flaps by PriA is required to activate origin-independent synthesis in cells lacking RNase HI, and whereas expression of yeast RNase H1 suppresses this origin-independent synthesis, it has hardly any effect in cells lacking RecG [13]. These findings do not exclude the possibility that RecG dissociates R-loops in vivo, but if it does the absence of this activity contributes little to the origin-independent synthesis observed in *recG* cells [13], which appears to stem almost exclusively from pathological events initiated in the terminus area of the chromosome.

## 6. Conclusions

We are just beginning to unravel the events associated with the fusion of two replication forks and the role the replication fork trap might have in this. However, an increasing number of experimental results suggest that a number of proteins such as RecG and 3’ exonucleases have an important function in preventing chromosome over-replication triggered by head-on fork fusion events, and it is significant that this over-replication in cells lacking RecG is blocked by mutations (*priA300*, *srgA1*) that also suppress many features of the *recG* mutant phenotype [100]. It indicates that this replication has pathological consequences. The need to avoid a destabilising effect on the genome may explain why RecG is conserved in almost all bacteria. It also provides an explanation for the importance for a replication fork trap in the termination area, and it might well explain why bacterial chromosomes are replicated from a single replication origin, as this limits the number of fork fusions. A chromosomal architecture with one defined replication origin and a distinct termination area, which per se defines two replichores, allows an uncomplicated way to not only coordinate co-orientation of replication and highly transcribed genes such as *rrn* operons, but also minimises the number of replication fork fusion events to exactly one per cell cycle. Thus, it appears that a variety of mechanisms are involved in preventing pathologies associated with replication fork fusion events, which would provide an explanation for the absence of a robust phenotype in cells in which the replication fork trap is inactivated. It requires modulation of several of these systems before distortions can be observed. Thus, the main function of the replication fork trap might not be to dictate where forks fuse, but to contain pathologies that can arise if fork fusion intermediates are not properly processed. It is tempting to speculate that the fork trap has evolved around the location where forks normally fuse, as suggested for the location of the *dif* site [58].

The idea that replication fork fusions can trigger re-replication of the already replicated DNA raises the question of what happens in archaea and eukaryotic cells. The single fork encounter experienced during normal replication of the *E. coli* chromosome contrasts sharply with the situation found in archaea and eukaryotes where there are many origins per chromosome, resulting in large numbers of fork collisions. In the archaeon *Sulfolobus solfataricus*, in which the circular chromosome is replicated from three origins, no clearly defined replication fork fusion points were observed, suggesting that replication termination occurs by random fusion of forks wherever they happen to meet. In addition, no correlation between termination and the *dif* dimer resolution site was found [101]. However, recent studies in eukaryotic cells have demonstrated that fork fusion events require a significant level of coordination [102,103,104]. In addition, defined proteins such as Rrm3 and Pfh1 helicases as well as the SCF^Dia2^ ubiquitin ligase have been associated with termination [105,106,107], and the increased genomic instability in ΔDia2 cells might indicate that the final stages of replication in eukaryotic cells has pathological potential [107]. RecG is absent from mammalian cells, but several studies have reflected on the ability of human and yeast helicases to remodel branched DNA structures in a manner reminiscent of RecG [108,109,110], and expression of *E. coli recG* partially suppresses the phenotype of human BLM-defective cell lines [111]. Indeed, limiting pathological events at termination may prove to be as crucial to genome stability as avoiding re-initiation at replication origins [112]. It certainly appears crucial for mitochondrial DNA replication. Human cells with specific defects of mitochondrial replication proteins show an accumulation of aberrant forked structures that is very reminiscent of the intermediates observed in *E. coli recG* cells [113,114].
